# 

**Published:** 1912-03-30

**Authors:** 


### BOOKS RECEIVED.

G. Gill and Sons, Ltd.: "First-air and Stretcher Drill," by D. M. MacDonald.

P. S. King and Son: "Report of Proceedings of the National Conference on the Prevention of Destitution."

J. Maclehose and Sons: "A Manual of Midwifery," by G. Balfour Marshall; "The Growth of Bone," by Sir Wm. Macewen, M.D.

J. B. Lippincott Co.: "Medical Diagnosis," by J. C Wilson; "The Surgery of Childhood," edited by Henry W Cattell, A.M., M.D.

Macmillan and Co., Ltd.: "Six Simple Talks on Health," by Helen G. Bowers.

### Appointments.

The following appointments have been made at the Glasgow Maternity and Women's Hospital: Dr. Gilbert J. Strachan and Dr. William Hamilton to be Indoor House Surgeons, and Dr. James Buchanan to be Outdoor House Surgeon.

Mr. C. A. Joll, F.R.C.S., has been appointed Senior Resident Medical Officer to the Royal Free Hospital for one year, to date from April 1, 1912.

Dr. Thomas Adam, M.D., M.B., Ch.B. Glasgow, has been appointed Medical Officer of Health for Stirlingshire. Dr. Adam's previous appointments include Assistantships at the Belvidere Fever Hospital and the Ruchill Fever Hospital, Glasgow.

Dr. A. M. Barford has been appointed Medical Officer of Health to Chichester.

Dr. W. Sibbold Scott has been appointed Medical Officer of Health for Ellesmere and district.

### Buildings Contemplated and in Progress.

Ayr.—The Ayr Town Council have issued instructions for the preparation of a design for a new diphtheria block to accommodate twelve patients.

Brentwood.—Alterations and additions are projected at the County and Borough Lunatic Asylum, Brentwood; plans may be inspected at the office of the county architect, Chelmsford.

Bristol.—Tenders are invited for a conversion of the Southmead Workhouse into an infirmary for Poor Law purposes. Particulars of the works, which are extensive, may be had from the architect, Mr. W. S. Skinner, of Bristol.

Brixen.—The *Zentral-Anzeiger,* Vienna, states that the Brixen municipal authorities have decided to erect an infirmary, at a cost of £63,700.

Burton-on-Trent.—The Guardians have adopted plans, prepared by Mr. J. Jenkins, for a children's receiving home at an estimated cost of £2,250.

Canterbury.—The Guardians have decided to build a laundry at an estimated cost, including machinery, of £2,000.

Carnarvon.—Tenders have been received for the Guardians' new infirmary, of which Mr. R. L. Jones, M.S.A., is the architect. The lowest of the nine tenders received amounts to £6,848 13s.

Croydon.—Tenders are invited for the erection of a boiler-house and chimney-shaft at the Waddon Borough Hospital. Particulars may be had from the borough engineer.

Hinckley.—Hospital plans are being forwarded to the Local Government Board for approval. The estimated cost is £5,110.

Holywell.—The Holywell Guardians have adopted plans for alterations to the workhouse premises at an estimated cost of £3,000.

Guisborough.—The Guardians have given instructions for the design of a new nurses' home at the workhouse.

London.—The Guardians of Mile End Old Town are about to consider tenders for a boiler-house extension, etc., at their workhouse, Mile End, E. Plans have been prepared by the architect to the Board, Mr. E. J. Harrison The contract contains a fair wages clause.

Nuneaton.—The Guardians invite tenders for the erection of a dining-hall and kitchen block at the Nuneaton Workhouse. Firms who desire to tender must apply to the architect, Mr. H. Quick, and tenders are to be opened on March 27.

Sheffield.—A competition, limited to local architects, is being held to ensure a good design for a cripples' home, to be erected in the New Rivlin Valley Road. Mr. E. M. Gibbs, F.R.I.B.A., is assessor in collaboration with the city architect, Mr. F. E. P. Edwards, F.R.I.B.A.

Wellington (Salop).—Mr. L. J. Moore, of London, is architect for a proposed hospital at Wellington, to cost £3,400.

Winchester.—Mr. H. C. H. Nisbett is architect for the cottage homes on the estate newly laid out for the Ecclesiastical Commissioners. The memorial stone has been laid of the first block, which includes eighty-two homes. Messrs. Wise and Lansdell are the contractors.

Workington.—An effort is being made to raise funds for the erection of an operating theatre at the Workington Infirmary; the estimated cost is £1,500.

Worthing.—The Town Council have decided to instal electricity for illuminating purposes at the Swandean Hospital.

Winchester.—Fifteen tenders have been received for the erection of a nurses' home and maternity wards at the Guardians' premises. The highest tender amounts to £2,784, and the lowest, which is accepted, to £2,510.

### Florence Nightingale Memorial.

The following contributions have been received by Mr. G. Q. Roberts since last week : The Hon. F. N. Curzon, £20; Corps of Military Police, Aldershot, £10 14s. 3d.; Officers of Headquarters' Staff, Aldershot, £8; Lady Blanche Haygarth, £5 5s.; Mrs. Pitt Draffen, £5 5s.; Denys Hague, Esq., £5; Edwin Bayley, Esq., £5; Frederick Lee, Esq., £5; the Rt. Hon. the Earl of Lytton, £5; Herbert H. Swift, Esq., £5; Miss Felix Smith, £3 3s.; Miss E. N. Paget, £2 2s.; J. Hamilton Houldsworth, Esq., £2 2s.; Mrs. Walter Druce, £2 2s.; Admiral Sir George Atkinson-Willes, £2 2s.; "A" Signal Co., Royal Engineers, £1 15s. 10d.; Nurse A. Roberts's Collection in Pwllheli, £1 8s. 9d.; Officers of 2nd Middlesex Regiment, £1 2s.; Mrs. William Coode, £1 1s.; Mrs. Jeffcock, £1 1s.; G. R. Fife, Esq., £1 1s.; Lady Sargood, £1 1s.; Miss Sargood, £1 1s.; the Hon. Ellinor and the Hon. Elizabeth Wilson-Patten, £1 1s.; Miss Katharine H. Greg, £1 1s.; Mary Lady Gerard, £1 1s.; Mrs. Rose Johnston, £1; Miss F. Arbuthnot, £1; Edward R. P. Moon, Esq., £1; the Rev. Canon J. H. Moore, £1; Col. G. Rowcroft, £1; N.C.O.s and men 1st Somerset Light Infantry, 17s. 10d.; Private Nurses' Institution, Royal Berks Hospital, Reading, 15s. 6d.; 5th Field Co., Royal Engineers, 14s. 3d.; D. J. Kennedy, Esq., 10s. 6d.; Miss E. Jex-Blake, 10s. 6d.; Mrs. Miller Morison, 10s.; Miss L. C. Johnson, 10s.; Mrs. Nott, 10s.

### Gifts and Bequests.

Mr. Wm. Hy. Liversidge, of Whyteleafe, Surrey, has left £300 to the Earlswood Asylum.

Mrs. Emily Fowler, of Worthing, has left £500 each to the Worthing Hospital and to the Cancer Hospital, Fulham Road.

Lord Donoughmore, Treasurer of the London Homœopathic Hospital, Great Ormond Street, W.C., has received £200 from R. Nivison, Esq., towards the general funds.

Mr. George Thos. Mackley, of Norwood, has left £100 each to the Eltham and Mottingham Cottage Hospital, the Surgical Aid Society, and to the Upper Norwood Cottage Hospital.

### The Week's Novelties.

### BOARD-ROOM FURNITURE AT TRADE DISCOUNT.

Institutional managers who, as members of the British Hospitals Association, are anxious to secure at a 33 per cent. trade discount new furniture for the quarters of their resident staffs or for their board room, should send a line to Hawkey and Gifford, 25 and 27 Curtain Road, London, E.C. A new catalogue has just been issued which contains special illustrations of those bedroom and sitting-room suites for which hospital committees are always in search of well made and economical examples. The designs are so varied that any type can be found.

### THE "WATERLEAP" FLANNEL WASHER.

This machine is not only known to hospitals, but ought to be known exceptionally well, as it has been alluded to before in these columns. The original principle of washing by water only is the basis of the contrivance which Messrs. R. G. Whitaker, Ltd., of London, have designed. The important point to institutional managers, however, is that the makers are willing to demonstrate to any secretary interested how "The Waterleap" makes damage of shrinkage to woollens impossible. On this no more need be said. This demonstration should not fail to convince all who witness it.

### THE WHISPERPHONE.

People who are in the habit of frequently using public telephones will welcome the neat little pocket mouthpiece called the "Whisperphone," which has been patented and placed on the market by the Whisperphone Syndicate, Limited, of 82 Fenchurch Street, E.C. Apart from the actual risk of approaching the mouth to a receiver which is still wet with the condensed moisture from the breath of a previous user, it is always repugnant to do so. This little apparatus is easily carried in the pocket, and can be applied to any telephone in a second. Another important advantage is that the Whisperphone enables the user to speak through the telephone in little more than a whisper and yet still be heard distinctly. This is an obvious advantage when privacy is desired and other persons are near at hand. The Whisperphone is fitted with an ingenious antiseptic chamber, is very easily cleaned, and has no parts which can get out of order. It is supplied in two styles at a cost of 2s. 6d. and 3s. 6d.

